# Effects of the transcutaneous electrode temperature on the accuracy of transcutaneous carbon dioxide tension

**DOI:** 10.3109/00365513.2011.590601

**Published:** 2011-07-06

**Authors:** Line C Sørensen, Lene Brage-Andersen, Gorm Greisen

**Affiliations:** 1 Department of Neonatology, Copenhagen University Hospital, Rigshospitalet, Copenhagen; 2 Department of Paediatrics and Neonatology, Copenhagen University Hospital, Hvidovre; 3 Faculty of Health Sciences, University of Copenhagen, Copenhagen, Denmark

**Keywords:** Infant: newborn, infant: premature, carbon dioxide, blood gas monitoring, transcutaneous, pulmonary ventilation

## Abstract

**Aim:**

The harmful effect of hypocapnia on the neonatal brain emphasizes the importance of monitoring arterial carbon dioxide tension (PaCO2). Transcutaneous monitoring of carbon dioxide (tcPCO2) reduces the need for arterial blood sampling. Drawbacks are high electrode temperature causing risks of skin burning. The aim was to determine the accuracy and precision of tcPCO2 at reduced electrode temperature.

**Methods:**

Forty newborns (GA 24.9-41.7) were included. Two tc-monitors were applied (TCM4, Radiometer, Copenhagen). Arterial blood gas sampling and monitoring of tcPCO2-level at different electrode temperatures was done simultaneously (39°C, 40°C, 41 °C, 42°C, 44°C). Difference of PaCO2-tcPCO2 was expressed as a percentage of the mean.

**Results:**

Mean PaCO2 was 5.8kPa [3,2; 7.9]. Bias (PaCO2 -tcPCO2) increased from 5% at 44°C to 17% at 39°C, but did not differ significantly between 41°C and 40°C. The precision of the tcPCO2 at each temperature ranged from +7-10%. After correction for the temperature-dependent over-reading, we found increasing PaCO2 — tcPCO2 difference with increasing PaCO2, approx. 2% pr. kPa increase of CO_2_. Only mild transient erythema was observed.

**Conclusion:**

A lower electrode temperature in tcPCO2-monitoring increases systematic overreading of the tc-electrode. However, in very preterm babies, monitoring at 40°C or 41°C is possible provided a bias correction of 12-15% is applied.

## Introduction

The harmful effects of hyperventilation and hypocapnia on the neonatal brain emphasize the importance of a stable arterial carbon dioxide tension (PaCO2) especially in mechanically ventilated newborns [1]. Transcutaneous monitoring (TCM) allows continuous monitoring of the transcutaneous carbon dioxide (tcPCO2) – a surrogate measure of the PaCO2. Hence, the need of arterial blood sampling is reduced.

The transcutaneous electrode conventionally is heated to 44°C to arterialize the capillary bed. The correlation between tcPCO2 and PaCO2 depends on the temperature in the capillary bed, the blood flow to the skin, and the local metabolic production of CO_2_. Drawbacks of the high electrode temperature are risk of skin burns [2,3]. The correlation between time and surface temperature in the causation of cutaneous burns has been known for a long time [4]. Early studies in preterm infants showed a direct correlation between the duration of electrode application and persistence of erythema; while the application time is shortened [2]. Besides disturbance of the neonate, relocation of the electrode gives unstable values during stabilization.

In the early era of transcutaneous monitoring, several studies were performed, improving the monitors in use today [5,6]. This also includes studies with monitoring at different electrode temperatures [7-9]. Because of the fragile skin of preterm infants a lower electrode temperature would be appreciated. This could reduce the risk of skin burns and prolong the application time. However, a recent study in adults examined reduced electrode temperature using the TCM4, Radiometer Medical ApS, Denmark [10]. Due to bias, the authors concluded that the electrode should be heated to at least 43°C to measure reliable tcPCO2 [10].

The aim of this study was to determine the accuracy and precision of tcPCO2 in preterm infants using TCM4, Radiometer Medical ApS, Denmark, with reduced electrode temperatures. Our assumption was that electrode temperatures of 39-41°C would be less damaging to the skin, since these temperatures are reached during systemic fever.

## Patients and methods

The study was conducted in the NICU at the Copenhagen University Hospital, Rigshospitalet. The local ethics committee approved the study (J. nr. (KF) 01 283061). Parental consent was obtained in all cases.

This paper consists of two studies. In the first study, pCO2 precision was examined at five different electrode temperatures (39, 40, 41, 42 and 44°C). Recruitment was from January to April 2006. Inclusion criteria were clinical indication of transcutaneous (TC) monitoring and presence of an umbilical or radial arterial catheter. A total of 20 newborns irrespective of gestational age (GA) were included (= GA mixed group).

In the second study, based on results from study one, comparisons of pCO2 precision at selected electrode temperatures were done (40, 41 and 44°C). Recruitment was from November 2006 to June 2008. Inclusions criteria was GA below 34 weeks or birth weight below 1500 g, clinical indication of TC monitoring and presence of an umbilical or radial arterial catheter. A total of 20 preterm infants were included (= Preterm group). Inclusion and examination was within the neonatal period (first 28 days of life).

All newborns were monitored using two TC monitors simultaneously (TCM 4 with combined CO_2_/O_2_ electrode E5280, Radiometer Medical ApS, Denmark). Because of higher values of pCO2 when elevating skin temperature (anaerobic factor 4.5%/°C), the CO_2_ production of epidermal cells ('metabolic’ constant) adding to the pCO2 measured transcutaneously, the monitor has a correction algorithm integrated [11]. We used standard recommendations with the anaerobic factor (Severinghaus constant) turned on, and with a metabolic constant set at 0.5 kPa.

**Figure 1:**
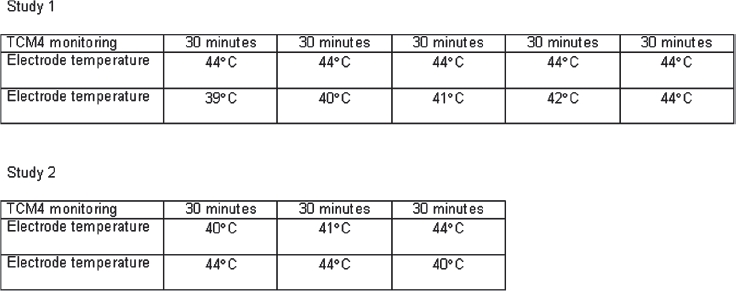
The sequence of the transcutaneous electrode temperature changes in the two studies.

Using two TCM4 simultaneously in the first study, allowed one electrode to remain unchanged at 44°C while the temperature of the other electrode was increased every half hour (39, 40, 41, 42 and 44°C) (Figure 1).

Using two TCM4 simultaneously in the second study allowed a cross over design to investigate the impact of preheating of the skin on pCO2 precision (Figure 1).

The TC electrodes were fixed to the skin of the trunk using a sticky fixation ring (E5260/E5280: Fixation ring 904 – 891; 30 mm; Radiometer Medical ApS, Denmark). The localization was not changed during the study. The electrode housing was 15 mm in diameter. After a few droplets of an electrolyte solution enhancing contact between electrode and skin, the electrode was fixed in the fixation ring (Electrolyte solution; Radiometer Medical ApS, Denmark).

The ‘gold standard’ to which the TC values were compared was the arterial pCO2 (PaCO2).

All arterial blood samples were taken from the indwelling catheter. Blood was sampled in heparinized capillary tubes (100 microliter). Immediately after sampling the tube was turned slowly 20 times. Within 5 min the blood gases were analysed using Blood Gas Analyzer ABL 735 (Radiometer Medical ApS, Denmark). In the second study, the first blood sample was analysed in duplicate to estimate test-retest variation of arterial pCO2.

Arterial blood gas sampling and readings of the tcPCO2-level was done simultaneously. After changing the electrode temperature, a minimum of 30 min. was allowed for temperature equilibration and for stabilizing tcPCO2 before blood sampling. Before measuring at a new electrode temperature, the sensor was calibrated. The monitor automatically performs this process using a built in calibration module and gas cylinder. Calibration of the sensor is recommended as a routine procedure in TC monitoring using TCM4. The sensor was re-membraned when needed (E5260/E5280: Membrane 904 – 892; Radiometer Medical ApS, Denmark).

Statistical analysis was done using SPSS version 12.0 (Chicago). To estimate the repeatability of the blood gas analysis we calculated the standard deviation of the differences between the pairs of blood gas measurements. Univariate ANOVA was used to test the influence of electrode temperature on accuracy (ΔtcPCO2 – PaCO2). 95% limits of agreement were calculated as described by Bland and Altman [12]. As the difference between transcutaneous and arterial pCO2 increased with increasing pCO2, the difference between transcutaneous and arterial values was expressed as a percentage of the mean (100 × (diff. tcPCO2 – PaCO2/mean tcPCO2 – PaCO2)). The Kruskal-Wallis test was used for comparisons.

## Results

The median gestational age in the GA mixed group was 31.1 weeks [24.9; 41.7] and median birth weight 1822 g [735; 3800 g] (Table I).

**Table I:** Group characteristics.

	GA mixed group (*N* = 20)	Preterm group (*N* = 20)
Gestational age (weeks)	31.1 [24.9; 41.7]	28.9 [25.6; 32.0]
BW (grams)	1822 [735; 3800]	1006 [590; 1900]
Nasal CPAP/Mechanical ventilation	50%/35%	55%/45%
No oxygen demand	55%	30%
Oxygen supplement (% O2)	21% [21%; 55%]	28% [21%; 70%]
Dopamine	0%	10%

Diagnoses were manifold: Prematurity (*n* = 11), asphyxia (*n* = 1), meconium aspiration syndrome (*n* = 1), necrotizing enterocolitis (*n* = 1), congenital heart disease (*n* = 3), oesophageal atresia (*n* = 1), extracorporeal membrane oxygenation (*n* = 1) and no diagnosis specified (*n* = 2).

In the preterm group, the median gestational age was 28.9 weeks [25.6; 32.0] and median birth weight 1006 g [590 g; 1900 g] (Table I). All infants (*n* = 20) were preterm, however other diagnoses included pneumothorax (*n* = 4), rhesus immunization (*n* = 1), persistent fetal circulation (*n* = 1) and lung bleeding (*n* = 1).

No infants were excluded due to instability, since this was not a part of the protocol. However, if there were any events that potentially could influence results, it was remarked. In the GA mixed group, two infants had remarks: ‘restless’ (*n* = 1) and ‘apnoea, bradycardia and desaturation of prematurity’ (*n* = 1), while in the preterm group four infants had remarks: ‘restless’ (*n* = 2), ‘increasing oxygen demand’ (*n* = 2), ‘apnoea, bradycardia and desaturation of prematurity’ (*n* = 4).

Arterial pCO2 (*n* = 220) was normally distributed (range 3.2-7.9; mean 5.8 kPa ± 1.0 kPa) and was not associated with electrode temperature (*p* = 0.9). The repeatability of arterial pCO2 measurements (*n* = 19) was 0.07 ± 0.15 kPa.

By defining a maximum of 1 kPa bias between tcPCO2 and PaCO2, we examined how many of the paired samples met these qualifications. When using an electrode temperature of 39°C 45% had a bias below 1 kPa (9/20 measurements), at 40°C 73% (44/60 measurements), at 41°C 68% (27/40 measurements), at 42°C 90% (18/20 measurements) and at 44°C 94% had a bias below 1 kPa (75/80 measurements).

**Figure 2:**
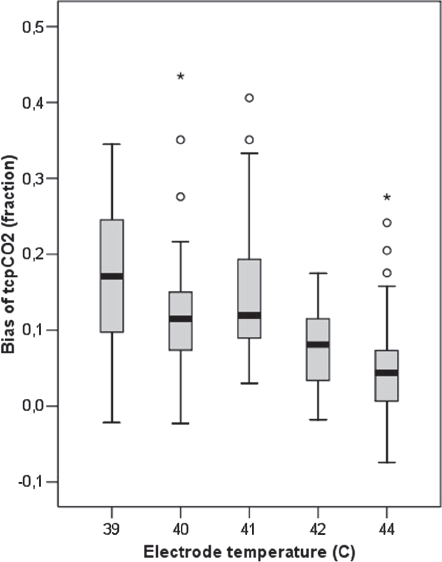
The difference between transcutaneous pCO2 and arterial pCO2 increases at lower electrode temperatures. The median difference is +12% at 40-41°C. Outliers also occur at high electrode temperature.

**Figure 3:**
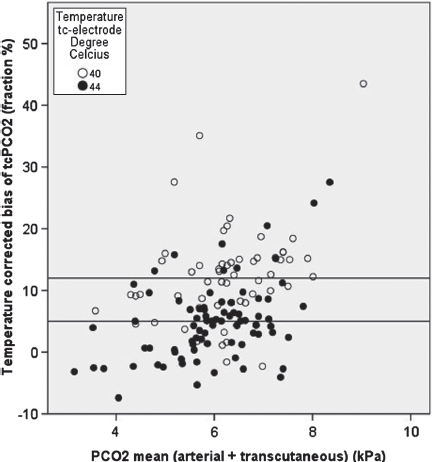
This figure shows the mean pCO2 (kPa) versus the temperature corrected bias of TcPCO2 (fraction, %). The horizontal lines show the mean bias of TcPCO2 at 40°C (12%) and 44°C (5%). Correction with 12-15% when using 40°C will approximate measurements to the arterial pCO2.

The mean PaCO2 - tcPCO2 difference (bias) increased from 5% at 44°C to 17% at 39°C, but did not differ significantly between 41°C and 40°C (median 14.8% vs. 11.8%) (Figures 2 & 3). The precision of the tcPCO2 at each temperature ranged from 7-10%. After correction for the temperature-dependent overreading, we found increasing PaCO2 -tcPCO2 difference with increasing PaCO2, an approx. 2% pr. kPa increase of pCO2.

All outliers were above the 95% confidence interval. These outliers occurred primarily at low electrode temperature. Sequence of electrode temperatures, including preheating of the skin to 44°C, had no influence on precision. We did not find any significant electrode drift after each session by control measurement using the calibration gas. No skin lesions apart from mild transient erythema were observed.

## Discussion

The aim of this study was to investigate how reduction of the electrode temperature influences accuracy and precision in tcPCO2 measurements in newborn infants. Lower electrode temperature reduces the risk of skin burning and allows longer periods of continued monitoring [13].

The bias of tcPCO2 using the normal electrode temperature of 44°C was approximately 5%. We had set the metabolic constant at 0.53 kPa (4 mmHg), which appears to be too low for our population.

Lower electrode temperatures were associated with higher bias, but at 41°C the bias was still typically less than 1 kPa. Precision was unchanged. We therefore conclude that it is reasonable to monitor tcPCO2 with a temperature of 41°C, particularly if the bias of 12-15% is accounted for. However, changing the electrode temperature requires an increased clinical awareness. With measurements close to the normal PaCO2 range an error of 1 kPa will have no great consequences, however readings in the subnormal range of PaCO2 can have a significant impact on cerebral perfusion. On our ward, we recommend an arterial blood gas if there is any doubt of the actual level of PaCO2 and in particular if the tcPCO2 is out of the normal range. At 41°C, the tcPO2 cannot be relied upon, because the capillary bed will not be sufficiently arterialized. The best procedure would be to deactivate the pO2 readout of the TCM4. In most situations, oxygen treatment of newborns can be sufficiently monitored by pulseoximetry.

Our results agree reasonably with previous studies. A study in neonates found a good correlation between tcPCO2 and arterial CO_2_ at 42°C and even 37°C [7]. In adults, a good correlation was found between transcutaneous and capillary pCO2 at 37, 39, 41, 43 and 45°C, provided the skin was preheated to 45°C [14]. The correlation coefficient, however, ignores bias. Fanconi and colleagues reduced the electrode temperature to 42°C and had reliable results, but only after topical metabolic inhibition [13]. Furthermore, after 12 h continuous use some neonates had skin burns [13]. In adults, the TOSCA with an electrode temperature of 42°C had a bias of +0.02 kPa, only [15]. The TOSCA monitor initially preheats the skin to 45°C for rapid arterialization before measurements and measurements were performed at the earlobe, using an ear clips. We did not find any effect of using high temperature before low temperature, or vice versa.

Surprisingly, in our study the bias was pCO2-dependent. The transcutaneous electrode uses an electrochemical principle: tcPCO2 is measured by determining the pH of an electrolyte layer separated from the skin by a highly permeable membrane. Diffusing CO_2_ changes the pH of the electrolyte solution and the pH changes are proportional to the logarithm of pCO2-changes. Since tissue heating increases tissue pCO2 by 4.5%/°C and some CO_2_ is produced locally, the tcPCO2 is higher than the arterial pCO2 and corrections for this are included in the monitor. For optimal use, the electrolyte and membrane must be changed every week, and the sensor must also be calibrated whenever a new patient is monitored. This was done meticulously for our two experimental monitors and furthermore control measurements using the calibration gas were done after measurements in all infants. If out of the acceptable range, recalibration and/or change of membrane was done. Therefore we cannot explain the pCO2 dependency of the bias. Combined with the upwards skew of the distribution of transcutaneous-arterial pCO2 difference, it meant that occasionally tcPCO2 overestimated significantly, and this occurred more often at high arterial tcPCO2. In practical terms this means that a very high tcPCO2 must be used with care. Safe practice would be taking a blood sample before significant clinical action, e.g. intubation for mechanical ventilation.

In conclusion, a lower transcutaneous electrode temperature increases the systematic overreading of tcPCO2. Monitoring at 40 or 41°C, however, appears reasonable since the typical bias is less than 1 kPa. In some cases with high arterial pCO2 the overreading was clinically significant and care should be taken that this does not have undue clinical consequences.
